# Development of Spray-Dried Micelles, Liposomes, and Solid Lipid Nanoparticles for Enhanced Stability

**DOI:** 10.3390/pharmaceutics17010122

**Published:** 2025-01-17

**Authors:** Shradha Dattani, Xiaoling Li, Charina Lampa, Amanda Barriscale, Behzad Damadzadeh, David Lechuga-Ballesteros, Bhaskara R. Jasti

**Affiliations:** 1 Department of Pharmaceutics and Medicinal Chemistry, University of the Pacific, Stockton, CA 95211, USA; p_dattani@u.pacific.edu (S.D.); xli@pacific.edu (X.L.); 2 Inhalation Product Development, PT&D AstraZeneca, LLC, South San Francisco, CA 94080, USA; c_lampa@u.pacific.edu (C.L.); amanda.barriscale@vaxcyte.com (A.B.); behzad.damadzadeh@gilead.com (B.D.); david.lechuga@astrazeneca.com (D.L.-B.)

**Keywords:** micelles, liposomes, solid lipid nanoparticles, LDV peptide, targeting, paclitaxel, spray drying, stability

## Abstract

**Objectives:** Micelles, liposomes, and solid lipid nanoparticles (SLNs) are promising drug delivery vehicles; however, poor aqueous stability requires post-processing drying methods for maintaining long-term stability. The objective of this study was to compare the potential of lipid-based micelles, liposomes, and SLNs for producing stable re-dispersible spray-dried powders with trehalose or a combination of trehalose and L-leucine. This study provides novel insights into the implementation of spray drying as a technique to enhance long-term stability for these lipid-based nanocarriers. **Methods:** Aqueous dispersions of LDV-targeted micelles, liposomes, and SLNs loaded with paclitaxel (PTX) were converted into re-dispersible powders using spray drying. The physicochemical properties of the nanocarriers were determined via scanning electron microscopy (SEM), Karl Fischer titration, differential scanning calorimetry (DSC), and dynamic light scattering (DLS). Short-term stability of all nanocarrier formulations was compared by measuring particle size, polydispersity index (PDI), and paclitaxel retention over 7 days at room temperature and at 4 °C. **Results:** Paclitaxel-loaded micelles, liposomes, and SLN formulations were successfully converted into well-dispersed spray-dried powders with acceptable yields (71.5 to 83.5%), low moisture content (<2%), and high transition temperatures (95.1 to 100.8 °C). SEM images revealed differences in morphology, where nanocarriers spray-dried with trehalose or a combination of trehalose and L-leucine produced smooth or corrugated particle surfaces, respectively. Reconstituted spray-dried nanocarriers maintained their nanosize and paclitaxel content over 7 days at 4 °C. **Conclusions:** The results of this study demonstrate the potential for the development of spray-dried lipid-based nanocarriers for long-term stability.

## 1. Introduction

Nanomedicines are established drug delivery vehicles that are well known for enhancing therapeutic efficacy, reducing toxicity, and promoting specific tumor accumulation through passive and active targeting mechanisms. Among all nanocarriers on the market or in clinical trials, lipid-based nanocarriers are in the most prevalent category, including micelles, liposomes, and SLNs [1,2]. Micelles are self-assembled amphiphilic nanostructures with a hydrophobic core and a hydrophilic shell, enabling the encapsulation of poorly water-soluble drugs. Their small size (10–100 nm) enhances tumor penetration via the enhanced permeability and retention (EPR) effect, improving drug delivery efficiency and accumulation at tumor sites, making them suitable for anti-cancer therapy [3]. Liposomes are nanocarriers composed of one or more phospholipid bilayers surrounding an aqueous core, enabling the encapsulation of both hydrophilic and hydrophobic drugs. They are widely utilized in drug delivery due to their biocompatibility and low toxicity [4]. SLNs are composed of a solid lipid matrix that remains solid at both room and body temperature, offering enhanced physical stability and controlled drug release. Like liposomes, they can encapsulate both hydrophilic and hydrophobic drugs; however, their solid lipid core forms a protective matrix around the encapsulated drug, reducing drug mobility and preventing premature release [5]. Moreover, micelles, liposomes, and SLNs can be surface-modified via PEGylation or targeting ligands to prolong circulation time, reduce systemic clearance, and enhance targeting efficacy. Despite these advantages, translation into a clinical product is faced with significant challenges, including poor encapsulation stability upon intravenous administration as well as physical and chemical instability during long-term storage when stored as an aqueous dispersion. Specifically, lipid-based nanocarrier solutions are known to undergo chemical degradation over time, via the oxidation of fatty acid chains and the hydrolysis of ester bonds, resulting in the generation of free fatty acids, lysophospholipids, and phospholgycerol compounds. Nanocarrier solutions can also become physically unstable during storage through processes such as vesicle fusion, aggregation, and drug leakage, resulting in potential safety concerns, the loss of therapeutic efficacy, and poor product quality [6]. For example, commercially available liposome products such as Doxil have exhibited poor stability in solution, including drug leakage and liposome aggregation [7]. Similarly, micelles often face limitations in maintaining their structural integrity during prolonged storage, which is linked to their dynamic nature [8]. In contrast, SLNs exhibit greater inherent stability due to their solid lipid matrix. However, they are susceptible to lipid polymorphic transitions during storage, resulting in drug release and aggregation, adversely affecting their physical stability and drug release profiles [9]. These limitations highlight the need for further research to investigate effective post-processing methods to maintain the long-term stability of these delivery systems. One such approach is for aqueous dispersions of nanocarriers to be converted into a dry powder that can be stored over a long period of time but can be reconstituted at the time of in vivo administration. As a result, stabilization is achieved by reducing water content and increasing the shelf-life. The drying method, critical process parameters, and formulation excipients require careful selection and optimization to ensure that dried nanocarriers can be re-dispersed without significant size changes, aggregation, or loss of drug content. Furthermore, dried powders should remain stable with low residual moisture content to prevent chemical degradation of the formulation components. Freeze drying is one of the most widely used techniques in the pharmaceutical industry for stabilizing nanocarriers and other drug formulations. Typically, nanocarriers are frozen in aqueous solution, followed by the removal of water from frozen samples by sublimation under vacuum. However, freeze drying is often associated with lengthy processing times, high energy consumption, and significant costs. Moreover, freezing and drying stresses can destabilize and disrupt lipid-based nanocarriers such as the liposome bilayer membrane structure due to the liquid–ice interface. This results in drug leakage, changes in size, aggregation, vesicle fusion, and ultimately poor reproducibility and scalability [10,11]. To address these limitations, spray drying offers several key advantages. Unlike freeze drying, spray drying operates as a single continuous-step process, significantly reducing processing times and energy consumption. Additionally, spray drying enables greater control over particle size, morphology, and surface characteristics, resulting in improved re-dispersibility and stability during storage. Importantly, rapid solvent evaporation in spray drying minimizes thermal and structural stresses on nanocarriers, preserving drug integrity and structural stability more effectively than freeze drying. These attributes collectively make spray drying a highly desirable approach for maintaining the long-term stability of nanocarrier solutions [12,13]. The process of spray drying nanocarriers into dry powders occurs in a single step via three stages; atomization, dehydration, and powder collection. Initially, the feedstock solution, which is the nanocarrier in aqueous solution, is atomized into a hot drying gas such as nitrogen. Atomization involves the application of an energy source that acts on a bulk liquid, resulting in the liquid’s break-up into individual spray droplets. Following atomization, microparticle formation occurs through the conversion of atomized spray droplets into solid particles. The solid particles are then separated from the process gas stream using a cyclone; this principle is based on the density difference between the particle and gas. To date, the main application of spray-dried powders has been for administration via inhalation for the pulmonary delivery of protein pharmaceuticals [13]. However, there are limited studies that have assessed spray drying as a technique for arresting drug passive diffusion and enhancing the long-term stability of re-dispersible nanocarrier formulations prior to intravenous administration. Therefore, evaluating the performance of micelles, liposomes, and SLNs as spray-dried formulations offers an opportunity to address existing limitations and identify the most suitable platform for clinical use. Furthermore, to the best of our knowledge, a comparative study evaluating lipid-based micelles, liposomes, and SLNs for conversion into spray-dried products has not yet been performed. In a previous study, LDV-targeted micelles, liposomes, and SLN solutions were compared for their encapsulation stability in vitro and in vivo, where SLNs demonstrated enhanced stability versus micelles and liposomes [14,15]. The current study seeks to further characterize the stability of these nanocarriers as spray-dried formulations to provide insights into the potential for scaling up, storage stability, and subsequent translation into clinical applications.

## 2. Materials and Methods

### 2.1. Materials

Peptide amphiphiles C16-(PEG2)_4_-LDV were synthesized at the University of the Pacific (Stockton, CA, USA) and by GenScript (Piscataway, NJ, USA). Solvents including HPLC-grade dichloromethane (DCM), Dimethylformamide (DMF), Acetonitrile, Methanol, Ethanol, and Chloroform were purchased from Fisherscientific (Pittsburgh, PA, USA). Piperidine, Trifluoroacetic acid (TFA), Triisopropyl silane (TIS), and Stearic acid were obtained from Acros organics (Fair Lawn, NJ, USA). DPPC lipids and a liposome extruder were purchased from Avanti polar lipids (Alabaster, AL, USA). Tripalmitin and D-(+)-Trehalose Dihydrate were purchased from Sigma-Aldrich (St Louis, MO, USA). Pierce Slide-A-Lyzer G2 dialysis cassettes (MWCO 10,000) were purchased from VWR (Visalia, CA, USA). L-leucine, aluminum stubs, and carbon adhesive tapes were provided by AstraZeneca (South San Francisco, CA, USA). Paclitaxel was purchased from LC laboratories, Woburn, MA, USA.

### 2.2. Preparation of LDV-Targeted Micelles, Liposomes, and SLNs

LDV-targeted micelles, liposomes, and SLNs of similar composition were prepared using palmitic acid-derived lipids previously described [14]. Briefly, peptide amphiphiles comprising two units of hydrophilic PEG2 linker units, LDV tripeptide, and palmitic acid (C16:0) were prepared using solid-phase peptide synthesis (SPPS) for synthesis of targeted nanocarriers. Peptide amphiphiles were purified by Reverse Phase High Performance Liquid Chromatography (RP-HPLC) using an Agilent 1200 HPLC (Santa Clara, CA, USA), lyophilized, and characterized by Electro Spray Ionization Mass Spectrometry (ESI-MS). Micelle formation from peptide amphiphiles was confirmed by assessing the Critical Micelle Concentration (CMC) using the pyrene fluorescence probe method, and LDV-targeted micelles were prepared by the thin-film method [14,16]. Paclitaxel and peptide amphiphiles were co-dissolved in methanol. Following solvent evaporation, thin films were rehydrated in aqueous medium to achieve a final peptide amphiphile concentration of 1 mg/mL and an initial drug load of 10 wt%. LDV-targeted liposomes were prepared using thin-film hydration and extrusion, using palmitic acid-derived double-chain amphiphilic phospholipid DPPC (16:0/16:0) for comparability with micelles [17]. Briefly, DPPC, peptide amphiphiles, and paclitaxel were dissolved in chloroform:methanol 2:1 (*v/v*), followed by solvent evaporation, hydration, freeze-thawing, and extrusion to achieve a final lipid concentration of 15 mg/mL and an initial drug load of 1 wt%. For SLN synthesis, a palmitic acid-derived, high-melting-point solid lipid known as tripalmitin (16:0/16:0/16:0) was selected for comparability with micelles and liposomes. SLNs were prepared via microfluidic mixing technology on the NanoAsssmblr (Benchtop, Precision NanoSystems Inc., Vancouver, BC, Canada) [18,19,20]. Mixtures of tripalmitin and paclitaxel in ethanol and peptide amphiphile in aqueous medium were introduced through a microfluidic cartridge at pre-determined flow rates, and the resulting mixtures were dialyzed to remove organic solvent, achieving a final lipid concentration of 1 mg/mL and an initial drug load of 10 wt%.

### 2.3. Preparation of Spray-Dried Micelles, Liposomes, and SLNs 

The feed solution was prepared by mixing aqueous solutions of PTX-micelles, PTX-liposomes, and PTX-SLNs with trehalose or a mixture of trehalose and L-leucine dissolved in purified water (Table 1). Trehalose and L-leucine excipients were selected for spray drying due to their stabilizing properties. Trehalose was used as a bulk-forming excipient, while L-leucine served as a stabilizer due to its surfactant-like properties [21]. The feed solution was fed into a custom-designed small-scale pharmaceutical spray dryer with a modified Buchi atomizer nozzle at a rate of 3 mL/min. The dryer inlet temperature was maintained at 60 °C to ensure efficient solvent evaporation while preventing thermal degradation of nanocarrier components. An outlet temperature of 50 °C was maintained below the glass transition temperature of trehalose to preserve particle stability. The drying gas flow rate was set at 850 slpm with an atomization gas flow rate of 15 slpm. The collector jacket and cyclone jacket temperatures were maintained at 50 °C. Sample handling was performed in a glove box with constant flushing of nitrogen, and the relative humidity was kept below 5% to protect the samples from moisture. Prior to characterization, spray-dried powder formulations were kept in a humidity-controlled cabinet at low relative humidity (≤5%).

### 2.4. Solid-State Characterization of Spray-Dried Powder Formulations

#### 2.4.1. Process Yields of Spray-Dried Powder Formulations

The process yields for all spray-dried PTX-micelles, PTX-liposomes, and PTX-SLNs were calculated from the mass ratio of the collected powders to the total solid content in the feed (Equation (1))

(1) $$\%\;yield=\frac{weight\;of\;spray\;dried\;powders}{total\;weight\;of\;solids\;added\;initially}\times 100$$

#### 2.4.2. Scanning Electron Microscopy (SEM)

The morphology of all spray-dried powder formulations was determined using a JSM-IT100 microscope (JOEL USA, Peabody, MA, USA), operating at an acceleration voltage of 20 kV. Spray-dried powder formulations for PTX-micelles, PTX-liposomes, and PTX-SLNs were deposited onto an aluminum stub coated with conductive carbon tape. The aluminum stubs coated with sample were then sputter-coated three times with gold-palladium for 60 s under high vacuum. SEM images were analyzed using image analysis software, version 1.

#### 2.4.3. Differential Scanning Calorimetry (DSC)

Thermal analyses for all spray-dried powder formulations of PTX-micelles, PTX-liposomes, and PTX-SLNs were performed using a differential scanning calorimeter (TA instruments DSC Q2000, New Castle, DE, USA). Approximately 15 mg of spray-dried powder formulations was loaded into an aluminum pan and hermetically sealed. Samples were heated at the scanning rate of 2 °C/min to 150 °C in a nitrogen atmosphere. All samples were prepared in triplicate and analyzed using the TA instrument explorer Qseries software, equipped with software version 24.11.

#### 2.4.4. Moisture Content

The residual moisture content of all spray-dried powder formulations of PTX-micelles, PTX-liposomes, and PTX-SLNs was determined by the oven vaporizer Karl Fisher coulometric titration method using a Metrohm 874 oven sample processor. The oven temperature was set to 150 °C, and the gas flow was set to 75 mL/min. Samples were prepared by adding 15 mg of spray-dried powder into vials and sealed with caps. A reference sample was also prepared by sealing an empty vial. All samples were prepared in triplicate and analyzed using Tiamo 2.5 software.

### 2.5. Physicochemical Characterization of Micelles, Liposomes, and SLNs

#### 2.5.1. Reconstitution of Spray-Dried Micelles, Liposomes, and SLNs

Spray-dried PTX-micelles, PTX-liposomes, and PTX-SLNs were reconstituted with purified water to achieve a final lipid concentration of 0.5 mg/mL. Nanocarrier solutions were agitated for approximately 5 min by hand until all the powder had dissolved. Syringe filtration was applied to nanocarrier solutions containing L-leucine, as these formulations did not fully dissolve in solution. 

#### 2.5.2. Size, Surface Charge, and Drug Content of Micelles, Liposomes, and SLNs

The physicochemical characteristics of PTX-liposomes and PTX-SLNs were determined before and after spray drying. Specifically, these nanocarriers were characterized for size, PDI, and surface charge in the feed solution and after rehydration post spray drying, using the mobius zeta potential and DLS detector (Wyatt, Santa Barbara, CA, USA). DLS measurements were not taken for PTX-micelles due to method incompatibility. The ratio of nanocarrier size before and after spray drying was determined using Equation (2), where *S_i_* and *S_f_* correspond to the initial and final sizes, respectively [22,23]. For the interpretation of these ratios, a value near 1 indicated preservation of size and acceptable colloidal stability during spray drying. Paclitaxel concentrations for PTX-micelles, PTX-liposomes, and PTX-SLNs were determined for all formulations using RP-HPLC as described above, and percentage recovery was calculated using Equation (3).

(2) $$size\;ratio=\frac{S_{I}}{S_{f}}$$

(3) $$\%\;paclitaxel\;recovery=\frac{initial\;amount\;ptx}{final\;amount\;ptx}\times 100$$

#### 2.5.3. Short-Term Stability of Reconstituted Micelles, Liposomes, and SLNs

Spray-dried PTX-micelles, PTX-liposomes, and PTX-SLNs were reconstituted in purified water and assessed for short-term stability over 7 days at room temperature and at 4 °C. As described above, the size and PDI were determined using the mobius DLS detector (Wyatt, Santa Barbara, CA, USA). Paclitaxel content was measured for all reconstituted spray-dried nanocarriers using RP-HPLC as previously described.

### 2.6. Statistical Analysis

Statistical analysis was performed using a paired t-test to compare the sizes of PTX-liposomes and PTX-SLNs before and after spray drying. A *p*-value of < 0.05 was considered statistically significant. All statistical analyses were performed with GraphPad Prism software, version 10.1.0.

## 3. Results

### 3.1. Spray-Drying Process Yield

The spray-drying process yield was determined for all spray-dried nanocarrier formulations. The results demonstrated process yields ranging between 71.5 to 83.5% for spray-dried PTX-micelles, PTX-liposomes, and PTX-SLN formulations (Table 2). There were no trends in process yields for nanocarriers spray-dried with trehalose versus those spray-dried with trehalose and L-leucine indicating that the choice of spray drying excipients did not impact the process yield. Comparatively, spray-dried PTX-micelle formulations produced lower process yields at 71.5 and 74.5% compared with PTX-liposomes and PTX-SLN formulations. Despite differences observed, the data indicate that the process parameters and formulation excipients were sufficient to produce adequate yields of spray-dried powder formulations.

### 3.2. Solid-State Characterization of Spray-Dried Powders

**Figure 1:**
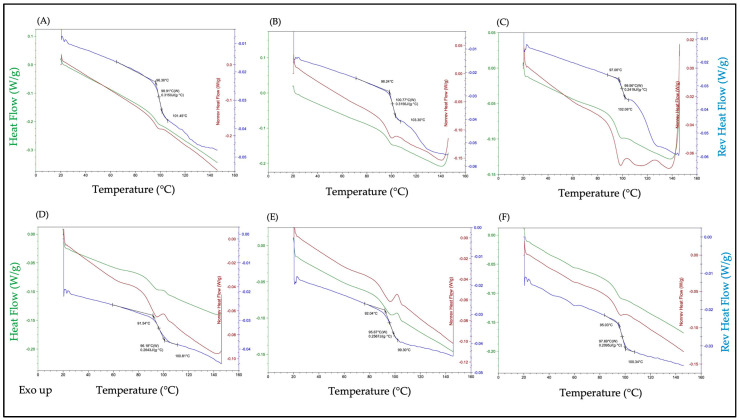
DSC thermograms for PTX-liposomes + trehalose (**A**), PTX-micelles + trehalose (**B**), PTX-SLNs + trehalose (**C**), PTX liposomes + trehalose + L-leucine (**D**), PTX-micelles + trehalose + L-leucine (**E**), and PTX-SLNs + trehalose + L-leucine (**F**). Each thermogram represents a single heating cycle, showing total heat flow (green), non-reversible heat flow (red), and reversible heat flow (blue) on the y axis.

**Figure 2:**
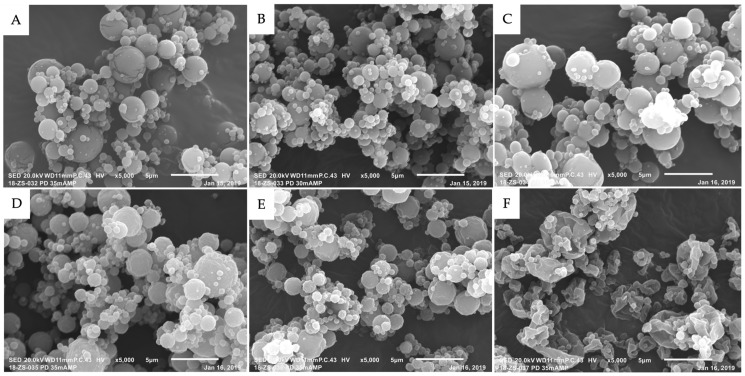
SEM images for PTX-liposomes + trehalose (**A**), PTX-micelles + trehalose (**B**), PTX-SLNs+ trehalose (**C**), PTX-liposomes + trehalose + L-leucine (**D**), PTX-micelles + trehalose + L-leucine (**E**), and PTX-SLNs + trehalose + L-leucine (**F**).

The glass transition temperature and hygroscopicity of carbohydrate excipients used for spray drying are important physicochemical properties that influence the physical stability of the final spray-dried powder product. Solid-state physicochemical characterization methods were carried out for all spray-dried powder formulations to assess physical stability. DSC results revealed similar thermograms for all spray-dried formulations, where mid-point transition temperatures ranging between 95.07 °C and 100.77 °C were observed (Figure 1, Table 2). These results were expected since the bulk of spray-dried powder formulations were composed of trehalose, and the measured transition temperatures corresponded with reported ranges [24]. The transition temperature is also a function of water content, and minimal changes across all spray-dried powder formulations indicated that changes in moisture content were also negligible. This was confirmed by Karl Fischer titration, which showed that the residual moisture content for all spray-dried powders was very low (<2%) and did not affect the transition temperatures of any spray-dried powder formulations (Table 2). Surface morphology was assessed by SEM, where images revealed smooth surfaces and spherical morphology of nanocarriers spray-dried with trehalose. Conversely, nanocarriers spray-dried with a mixture of trehalose and L-leucine produced corrugated particles with wrinkled morphology (Figure 2). 

### 3.3. Stability of Reconstituted Micelles, Liposomes, and SLNs

**Figure 3:**
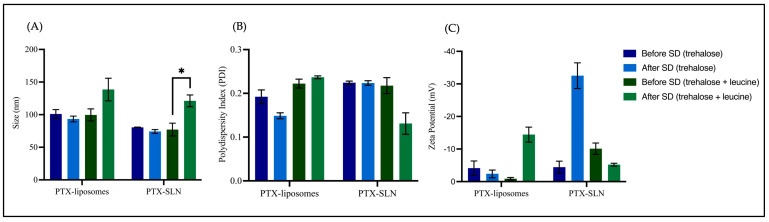
Graphs showing the mean size (**A**), PDI (**B**), and zeta potential (**C**) of PTX-liposomes + trehalose, PTX-SLNs + trehalose, PTX-liposomes + trehalose + L-leucine, and PTX-SLNs + trehalose + L-leucine, before and after spray drying. * Statistical significance was determined using a paired t-test, with *p* values < 0.05 indicating significant differences.

The size characterization results revealed that prior to spray drying, the colloidal stability of PTX-liposomes and PTX-SLNs was maintained in the presence of spray-drying excipients, where sizes ranged between 77.13 and 101.07 nm (Figure 3). To assess colloidal stability after spray drying, formulations were first re-dispersed in purified water, where nanocarriers spray-dried with trehalose were quickly reconstituted within a few minutes. However, the incorporation of L-leucine influenced dispersibility since these spray-dried powders did not dissolve completely upon reconstitution and required syringe filtration to remove undissolved particles. The results demonstrated that the sizes of PTX-liposomes decreased from 101.07 to 93.33 nm and the PDI decreased from 0.192 to 0.149 after spray drying with trehalose. The same trend was observed for PTX-SLNs, which showed a decrease in size from 80.53 nm to 74.20 nm after spray drying with trehalose (Table 3, Figure 3). Conversely, spray-dried formulations comprising a mixture of trehalose and L-leucine demonstrated increases in the sizes of PTX-liposomes (99.60 nm to 138.53 nm) and PTX-SLNs (77.13 nm to 121.20 nm) upon rehydration (Figure 3, Table 3), where an increase in size for PTX-SLNs was statistically significant (*p* = 0.04). The ratios of sizes before and after spray drying were determined, where PTX-liposomes and PTX-SLNs with trehalose revealed ratios close to one, indicating preservation of nanocarrier size after rehydration. For nanocarriers spray-dried with trehalose and L-leucine, ratios deviated from one, indicating that the spray-drying process impacted the sizes of PTX-liposomes and PTX-SLNs after rehydration. For surface charge evaluation, changes in the zeta potential of PTX-liposomes and PTX-SLNs were observed, indicating that the excipients or spray-drying process influenced the surface charge of these nanocarriers (Figure 3). With respect to drug content, PTX-micelles, PTX-liposomes, and PTX-SLNs retained > 60% of paclitaxel after spray drying, demonstrating that these formulations contained sufficient protectant to preserve the integrity of these nanocarriers (Table 3).

### 3.4. Short-Term Stability of Micelles, Liposomes, and SLNs

**Figure 4:**
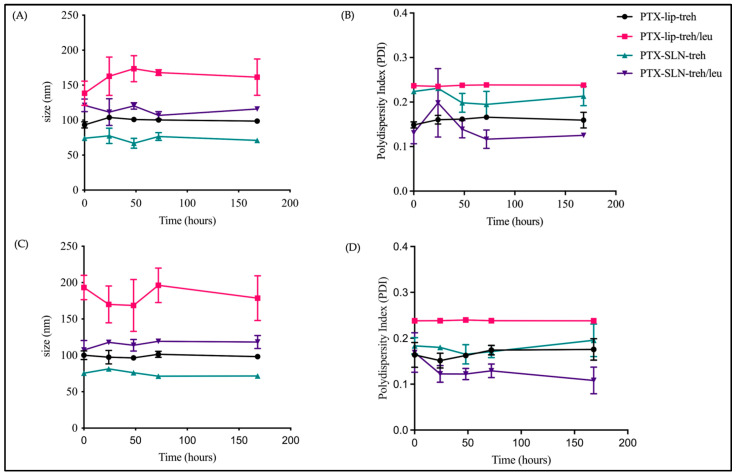
Short-term stability study. Graphs showing the mean size and PDI over 7 days at 4 °C (**A**,**B**) and at room temperature (**C**,**D**) for PTX-liposomes + trehalose, PTX-SLNs + trehalose, PTX-liposomes + trehalose + L-leucine, and PTX-SLNs + trehalose + L-leucine.

**Figure 5:**
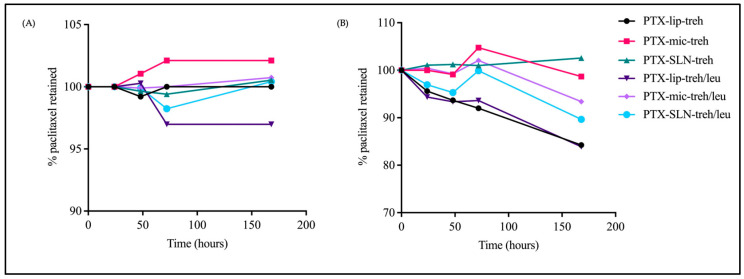
Short-term stability study. Graphs showing paclitaxel content at 4 °C (**A**) and at room temperature (**B**) over a period of 7 days for PTX-liposomes + trehalose, PTX-micelles + trehalose, PTX-SLNs + trehalose, PTX-liposomes + trehalose + L-leucine, PTX-micelles + trehalose + L-Leucine, and PTX-SLNs + trehalose + L-leucine.

The short-term stability of spray-dried PTX-micelles, PTX-liposomes, and PTX-SLNs was assessed by measuring physicochemical characteristics and paclitaxel retention over 7 days at 4 °C and at room temperature. The results showed that the sizes of PTX-SLNs spray-dried with trehalose or trehalose and L-leucine remained consistent over 7 days at room temperature and at 4 °C (Figure 4). The sizes of PTX-liposomes spray-dried with trehalose also remained consistent over time; however, PTX-liposomes with L-leucine demonstrated larger sizes and higher PDI values and a higher level of variability in size over time at room temperature and at 4 °C. This result indicates that spray drying with L-leucine impacted the physical stability of PTX-liposomes, which was further demonstrated by the reduction in paclitaxel content to 83.88% and 96.98% at room temperature and 4 °C at 7 days, respectively. Overall, for nanocarriers stored at 4 °C, paclitaxel content remained above 98% (except for PTX-liposomes with trehalose and L-leucine), demonstrating acceptable stability over 7 days. Interestingly, PTX-SLNs with trehalose were the most stable, as paclitaxel content remained consistent at approximately 100% for up to 7 days at room temperature (Figure 5).

## 4. Discussion

Lipid-based nanocarriers can undergo destabilization during storage, producing physical changes such as an increase in size, aggregation, and membrane fusion resulting in significant encapsulated drug leakage. Specifically, liposomes have been reported to undergo increased levels of aggregation during storage a few months post preparation, highlighting the necessity to produce stable powdered formulations more suitable for long-term storage [25]. Therefore, the conversion of nanocarrier dispersions to a powder form by spray drying is highly desired to enhance long-term stability. However, during the spray-drying process, heat and high shearing forces can result in the degradation of lipid components; therefore, optimization of formulation and process parameters is critical for maximizing process yield, preventing the loss of encapsulated drug, and maintaining the integrity of nanocarrier structure during dehydration. Typically, carbohydrate excipients such as mannitol, sucrose, or trehalose are added to the feed to serve as bulking agents or protectants that result in the formation of microparticles embedded with nanocarriers [17,26,27]. Disaccharides with high glass transition temperatures are desirable for spray drying to minimize the production of unstable hygroscopic powders prone to agglomeration. Maintaining moisture content at acceptable levels (<2%) is also critical since water acts as a plasticizer, causing mobilization of amorphous content, resulting in decreased transition temperatures of spray-dried powders. Other excipients such as amino acids and peptide sequences have also proved useful in spray drying by protecting against thermal stresses and denaturation, providing stabilization against aggregation and oxidation, and reducing the hygroscopicity of various formulations [21,28,29,30]. In this study, LDV-targeted PTX-micelles, PTX-liposomes, and PTX-SLNs were spray-dried with trehalose due to its high glass transition temperature and low hygroscopicity, which are essential for maintaining particle integrity and minimizing moisture-induced instability. The impact of L-leucine was also investigated, due to its surfactant-like properties, enabling migration to the surface of particles during the drying process. This process results in particle stabilization, reduced aggregation, and improved powder flow properties [21]. Additionally, the choice of critical process parameters is also dependent on formulation excipients. For example, the inlet temperature was selected to ensure efficient solvent evaporation while avoiding thermal degradation of nanocarrier components, such as LDV peptides, lipids components, and paclitaxel. Maintaining the outlet temperature below the transition temperature of trehalose ensured that the resulting powders remained physically stable with acceptable process yields. All aqueous nanocarrier dispersions were successfully converted into spray-dried powders with low moisture content (<2%), high transition temperatures, and high process yields (>70%), indicating that the spray-drying parameters selected were optimum for achieving stable dry powders. SEM images revealed differences in the morphology of nanocarriers, where formulations with trehalose or trehalose and L-leucine produced particles with smooth and corrugated surfaces, respectively (Figure 2). These differences did not impact the physical stability of spray-dried powders, as the absence of aggregates or fused particles indicated successful atomization of all nanocarrier formulations. Furthermore, the absence of particle fusion is essential for ensuring particle stability during storage and reconstitution. The differences in morphology observed can be attributed to the behavior of spray-drying excipients during the drying process. For example, smooth-surfaced particles were observed, as highly water-soluble excipients such as trehalose undergo continual shrinking as liquid droplets dry, leading to the formation of particles with smooth surfaces. These particles facilitated efficient water penetration and rapid reconstitution of all nanocarrier formulations upon rehydration. Conversely, corrugated particles can be attributed to the low water solubility of L-leucine, which precipitates out earlier in the drying process, resulting in formation of a solid shell that collapses later in the drying process [28]. While corrugated morphology enhances powder flowability and reduces adhesion, the hydrophobic nature of L-leucine may have hindered effective water penetration and particle wetting, creating barriers to efficient reconstitution. Similar findings have been reported in the literature, where L-leucine incorporation into spray-dried nano-formulations increased surface rugosity and subsequently impacted re-dispersibility and rehydration efficiency [31]. The physicochemical characterization of aqueous nanocarriers upon reconstitution is an important aspect of assessing the stability of nanocarriers, since the delivery of chemotherapeutic drugs is influenced by the physicochemical properties of nanocarriers, including their size and surface charge. For example, nanocarrier sizes ranging from 10 nm to 100 nm are ideal for tumor penetration and for avoiding renal clearance [32]. Additionally, the surface charge of nanocarriers should be neutral or negatively charged to minimize renal clearance, reduce cytotoxicity, prevent interactions with opsonins, and increase the circulation time [33]. Therefore, it is critical for spray-dried nanocarriers to maintain optimal physicochemical characteristics after reconstitution, as these parameters contribute to the efficacy of anticancer therapy. PTX-liposomes and PTX-SLNs were assessed for their potential to maintain optimal physicochemical characteristics, post spray drying, suitable for tumor targeting, where the size, PDI, and surface charge of nanocarriers were compared before and after spray drying. DLS measurements for micelles were not determined due to insufficient measurements and challenges in distinguishing the particle size distributions, indicating that the DLS method was not suitable for these micelle formulations. The limitations of the DLS method in accurately determining the size of PTX-micelles was likely due to their dynamic and polydisperse nature, which led to insufficient measurements in our study. Alternative techniques, such as transmission electron microscopy (TEM), cryogenic-TEM and small angle X-ray scattering (SAXS), may provide valuable information on micelle size and morphology. Cryo-TEM enables direct visualization of micelles in their native hydrated state, while SAXS provides detailed insights into structural features and size distribution [34]. In a previous study, Cryo-TEM successfully confirmed the presence of micelles that were not subjected to spray drying [14]. Future research could leverage these techniques to further characterize micelle formulations after spray drying, addressing limitations encountered with DLS. Overall, the choice of excipients impacted the size of PTX-liposome and PTX-SLN nanocarriers, where trehalose resulted in decreased sizes post spray drying. Decreases in particle sizes have previously been observed and may be due to the behavior of trehalose during the drying process and adsorption onto the nanocarrier surface [35]. Conversely, spray drying with L-leucine resulted in larger sizes, which has previously been observed where concentrations of L-leucine at 1% (*w*/*w*) increased the size of liposomes significantly after spray drying. These changes in particle size may be due to the hydrophobic nature of L-leucine and subsequent partitioning into the membrane of lipid vesicles during the drying process [21]. Despite the changes in size upon rehydration, the sizes of PTX-liposomes and PTX-SLNs remained below 200 nm, which is acceptable for tumor targeting and drug delivery. Furthermore, surface charge characterization results demonstrated that the overall surface charge of PTX-liposome and PTX-SLN nanocarriers remained negative or close to neutral, which is optimum for decreased aggregation and interactions with blood components such as opsonins [36]. The efficiency of the spray-drying process was further demonstrated by drug retention characterization post spray drying, where PTX-micelles, PTX-liposomes, and PTX-SLNs retained high amounts of paclitaxel (>60%), indicating that these formulations contained sufficient protectant to preserve the integrity of these nanocarriers. These nanocarriers were further characterized and compared for their potential to remain stable in aqueous solution during a short-term stability study. The results demonstrated that all reconstituted aqueous formulations were physically stable for up to 7 days at 4 °C. Furthermore, formulations that had greater stability at room temperature were PTX-SLNs and PTX-micelles containing trehalose, whereas their respective formulations containing L-leucine were less stable in terms of paclitaxel content. These results, along with the observed increase in nanocarrier size in formulations containing L-leucine, suggests that the partitioning of L-leucine into the lipid nanostructure may have disrupted the lipid nanostructure, resulting in destabilization and drug release from these nanocarriers. Similar findings have reported an adverse impact of L-leucine on spray-dried formulations where the presence of higher L-leucine concentrations (>0.5% *w*/*w*) in spray-dried liposome formulations resulted in a significant increase in particle size and PDI upon rehydration [21]. Another study investigated the effect of L-leucine concentrations (0–50 wt%) on spray-dried viral vector vaccines for pulmonary delivery. The authors reported a decline in the bioactivity and aggregation of adenovirus particles as a result of interactions between L-leucine and viral vectors in the feed solution [37]. These findings underscore the importance of optimizing the L-leucine concentration to prevent destabilization and aggregation in spray-dried nanocarrier systems. Future investigations examining the distribution of L-leucine within the nanocarriers and its interaction with lipid components could provide deeper insights into this destabilization mechanism. Interestingly, PTX-liposomes both with trehalose and a combination of trehalose and L-leucine exhibited lower stability at room temperature in terms of paclitaxel retention, which can be attributed to structural changes, including aggregation and drug leakage, phenomena commonly observed among liposomes [7,38,39]. While direct comparisons between lipid-based SLNs, micelles, and liposomes remain limited, previous studies have demonstrated the effectiveness of spray drying in producing stable SLN formulations [29,40,41]. The findings from this study further support the suitability of spray drying SLNs, owing to their solid lipid matrix, which enables them to withstand the thermal and mechanical stresses applied during the spray-drying process.

## 5. Conclusions

Lipid-based nanocarriers are promising drug delivery vehicles for anticancer therapy. However, nanocarriers stored as aqueous solutions commonly result in chemical and physical instabilities, which is a significant hurdle for clinical use and commercialization. Therefore, the selection of suitable drying methods is critical for arresting drug passive diffusion and enhancing long-term stability. In this study, LDV-targeted PTX-micelles, PTX-liposomes, and PTX-SLNs were assessed for their potential to produce stable spray-dried powders that can be reconstituted prior to patient administration. All nanocarrier dispersions were successfully spray-dried, producing powders with low moisture content and high transition temperatures. For short-term stability assessment, the sizes of PTX-micelles were not determined due to incompatibility with characterization methods; therefore, drug retention was used to compare stability with PTX-liposomes and PTX-SLNs. Upon reconstitution, nanocarriers spray-dried with trehalose demonstrated acceptable drug retention and colloidal stability within the ideal size range for tumor targeting (10 to 100 nm), indicating that the spray-drying process parameters and formulation excipients selected were optimum for achieving reconstitutable stable dry powders. However, the incorporation of L-leucine in spray-dried formulations impacted reconstitution and nanocarrier size (>100 nm), as well as paclitaxel retention, indicating that these formulations were less stable compared to nanocarriers spray-dried with trehalose. The observed limitations suggest that further optimization of L-leucine concentrations within these nanocarrier formulations may be necessary to achieve improved stability. When comparing the stability of these nanocarrier systems, PTX-SLNs spray-dried with trehalose demonstrated good stability due to minimal changes in size and paclitaxel content under both 4 °C and ambient temperature storage conditions. These findings support the translational potential of PTX-SLN formulations as viable candidates for clinical applications, where stability during long-term storage and reconstitution are critical for therapeutic efficacy. Importantly, the spray-drying process is scalable, offering a cost-effective and efficient manufacturing pathway suitable for large-scale pharmaceutical production.

## Figures and Tables

**Table 1:** Composition of spray-dried nanocarrier formulations.

Nanocarrier	Nanocarrier (wt %)	Trehalose (wt %)	L-Leucine (wt %)	Concentration Feedstock (mg/mL)
**PTX-micelle**	2.5	97.5	-	20
**PTX-liposome**	2.5	97.5	-	20
**PTX-SLN**	2.5	97.5	-	20
**PTX-micelle**	2.5	77.5	20	20
**PTX-liposome**	2.5	77.5	20	20
**PTX-SLN**	2.5	77.5	20	20

**Table 2:** Process yield, transition temperatures, and moisture content of PTX-liposomes, PTX-micelles, and PTX-SLNs spray-dried with trehalose or trehalose and L-leucine.

Nanocarrier	Spray Drying Excipients	Process Yield (%)	Tg Onset (°C)	Mid-Point Tg (°C)	Moisture Content (%)
**PTX-micelle**	trehalose	71.50	97.65 ± 0.83	100.77 ± 0.00	1.29 ± 0.05
**PTX-liposome**	trehalose	79.50	94.26 ± 2.97	98.91 ± 0.00	1.85 ± 0.01
**PTX-SLN**	trehalose	80.50	96.33 ± 0.02	98.10 ± 0.10	1.42 ± 0.01
**PTX-micelle**	trehalose/L-leucine	74.50	92.11 ± 0.09	95.07 ± 0.86	1.08 ± 0.03
**PTX-liposome**	trehalose/L-leucine	83.50	90.98 ± 0.80	95.17 ± 1.43	1.44 ± 0.04
**PTX-SLN**	trehalose/L-leucine	79.50	95.39 ± 0.77	98.23 ± 0.77	1.01 ± 0.03

**Table 3:** Table showing observations, paclitaxel recoveries, and size ratios for PTX-liposomes, PTX-micelles, and PTX-SLNs spray-dried with trehalose or trehalose and L-leucine.

Nanocarrier	Spray-Drying Excipients	Observation upon Reconstitution	PTX Recovery (%)	Ratio (*S_i_*/*S_f_*)
**PTX-micelle**	trehalose	clear solution	98.79	ND
**PTX-liposome**	trehalose	clear solution	96.74	1.08
**PTX-SLN**	trehalose	clear solution	70.58	1.09
**PTX-micelle**	Trehalose/L-leucine	cloudy solution	96.08	ND
**PTX-liposome**	Trehalose/L-leucine	cloudy solution	88.27	0.72
**PTX-SLN**	Trehalose/L-leucine	cloudy solution	65.85	0.64

## Data Availability

The original contributions presented in this study are included in the article. Further inquiries can be directed to the corresponding author.
